# Linking cognitive functioning and postural balance control through virtual reality environmental manipulations

**DOI:** 10.3389/fnagi.2022.954050

**Published:** 2022-09-01

**Authors:** Yu Imaoka, Laura Hauri, Andri Flury, Eling D. de Bruin

**Affiliations:** ^1^Motor Control and Learning Laboratory, Institute of Human Movement Sciences and Sport, Department of Health Sciences and Technology, ETH Zurich, Zurich, Switzerland; ^2^Division of Physiotherapy, Department of Neurobiology, Care Sciences and Society, Karolinska Institute, Stockholm, Sweden; ^3^School of Health Professions, Eastern Switzerland University of Applied Sciences, St.Gallen, Switzerland

**Keywords:** posture, saccade, dementia, head-mounted display virtual reality technology, dual task, vestibular, cognitive impairment, cognitive function

## Abstract

**Background:**

Dementia is becoming a relevant problem worldwide. A simple screening at an early stage will be important to detect the risk of developing dementia. Vestibular dysfunction is likely to be associated with cognitive impairment. Since head-mounted display (HMD) virtual reality (VR) technology has the potential to activate the vestibular function, assessing postural sway with visual stimulation using HMD VR technology could be potentially useful for dementia screening.

**Objective:**

The purpose of this study is to evaluate the effect of HMD-based VR visual stimuli on posture in older adults and the relationship between the stimulated body sway behaviors and cognitive performance.

**Method:**

Using a cross-sectional study design, we investigated the effect of an optokinetic design-based room with stripes (OKR) VR environment oscillating forwards and backwards at 23/60Hz. Center of pressure (COP) displacement was measured in older adults aged 65 years and over in the OKR VR environment. The frequency response of COP was compared to the cognitive performance of the Montreal Cognitive Assessment (MoCA).

**Results:**

20 healthy older adults (70.4 ± 4.9 years; 27.2 ± 1.6 MoCA score) and 3 people with mild cognitive impairment (74.7 ± 4.0 years; 20.3 ± 2.1 MoCA score) were assessed. The results reveal that the oscillating OKR VR environment induced different postural sway in the anterior-posterior direction in the real world. Correlation analysis shows that the cognitive test score was associated with the frequency response of stimulated postural sway in the anterior-posterior direction (frequency Band 1 of 0−0.5Hz related to the visual and vestibular systems: *r*_*s*_ = 0.45, *P* = 0.03).

**Conclusion:**

Outcomes would suggest that a potential link may emerge between cognition and posture when the HMD-based VR visual stimuli are applied. The simple screening of stimulated postural sway could explain cognitive functioning. Further studies are warranted to clarify the vestibular system and spatial cognitive function more specifically in the proposed assessment system.

## 1. Introduction

As our society is aging, a burgeoning older population is at risk of developing dementia. Worldwide 50 million people lived with dementia in 2018 and the number is expected to rise to 152 million by 2050 (Patterson, 2018). While research has been undertaken to cure the disease, effective treatment has not been achieved (Nichols, 2019). However, growing evidence has shown that dementia may be preventable and, thus, early screening becomes more important (Robinson et al., 2015; Laver et al., 2016; Livingston et al., 2020).

**Figure 1:**
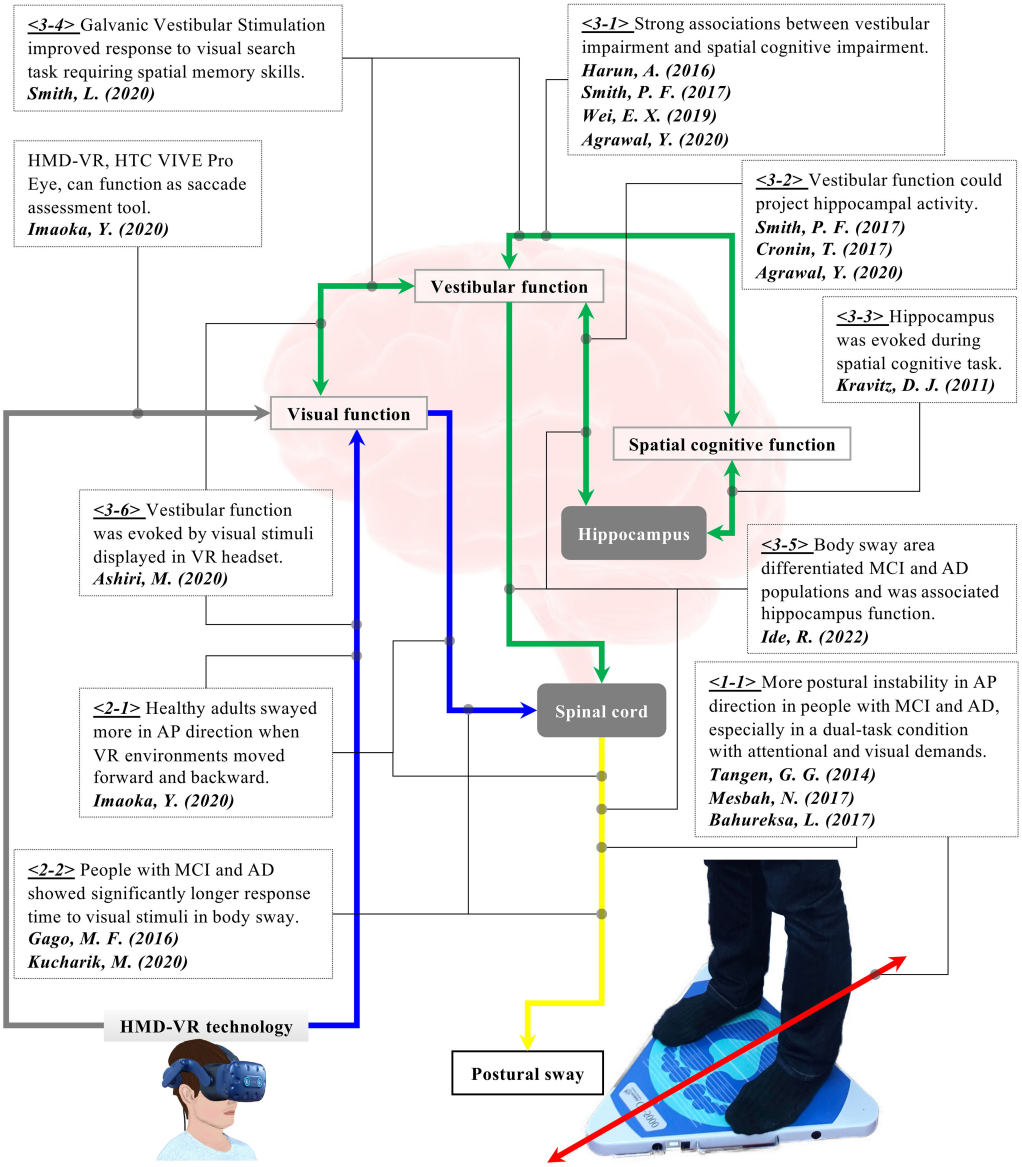
Research background for the hypothesis of the present study. <1-1> People with AD and MCI showed more postural instability in the anterior-posterior direction, especially in a dual-task condition with attentional and visual demands. <2-1> Visual flow in VR affected body sway in healthy older adults. <2-2> People with AD and MCI took a longer response time to visual stimuli. <3-1> Vestibular impairment was strongly associated with cognitive impairment in people with AD and MCI. <3-2, 3-3> Vestibular function could be linked to spatial cognitive function. <3-5> Postural sway was associated with the hippocampus and could differentiate people with MCI and AD. <3-4, 3-6> Visual stimuli displayed in HMD VR environments provoked activities in the vestibular function. Stimulation of vestibular function enhanced spatial memory skills. We have hypothesized that the cognitive function could be detected through postural sway behaviors when visual stimuli are applied in VR-HMD because VR visual stimuli can provoke the vestibular function that is potentially linked to the spatial cognitive function.

Among diverse assessment approaches for dementia screening, a biomechanical approach could be potentially useful. Prior studies explored important factors for mobility assessment of dementia, concluding that gait and posture were amongst the critical and feasible items (Trautwein et al., 2019; Van Ooteghem et al., 2019). In fact, it is reported that postural stability especially in the anterior-posterior (AP) direction significantly declined in people with mild cognitive impairment (MCI) and Alzheimer's Disease (AD) compared to healthy older adults (HA) (refer to <1-1> and corresponding red and yellow arrows in Figure 1). A study analyzing postural sway behaviors of people with AD also found that attentional demand during dual-task activity and visual challenge contributed to the postural instability of the AD population (Bahureksa et al., 2017; Mesbah et al., 2017). With the aim of improving body balance assessment for dementia screening using a visual challenge, we have created a new assessment system combining a stabilometer and head-mounted display (HMD)-based virtual reality (VR) technology (Imaoka et al., 2020a). The new system was developed to trigger postural sway in anterior-posterior direction intentionally by giving a visual flow of moving VR environments forwards and backwards. The study assumed that the provoked postural sway in the anterior-posterior direction could be a good indicator to classify people with dementia. The results showed that both healthy older and young adults swayed more in the anterior-posterior direction when they were exposed to the moving VR environments compared to the baseline eyes-open (EO) condition without the VR headset (refer to <2-1> with corresponding blue and yellow arrows in Figure 1). Other studies also induced postural sway by giving various visual stimuli to healthy older adults and people suffering from MCI and AD (Gago et al., 2016; Kucharik et al., 2020). The studies revealed that the population with MCI and AD took longer reaction times to the stimuli and swayed more compared to the healthy population (refer to <2-2> with corresponding blue and yellow arrows in Figure 1). These results imply that people with cognitive impairment possibly have dysfunction in adapting to moving visual stimuli. Therefore, visual stimulation from HMD-based VR can be a useful tool to elicit different postural sway behaviors, which could contribute to improving dementia screening (Liu et al., 2019). Nonetheless, studies suggest that a more hypothesis-driven approach should be included focusing on specific cognitive processes of people with dementia for a more reliable diagnosis (Sharples et al., 2008; Clay et al., 2020). Therefore, it is important to design VR environments that integrate cognitive challenges and trigger the relevant postural reactions.

An increasing number of studies have reported the possible relationship between the vestibular and cognitive functions. For example, research indicated a connection between vestibular dysfunction and cognitive impairment in people with AD (Harun et al., 2016; Wei et al., 2019; Agrawal et al., 2020) (refer to <3-1> with corresponding green arrow in Figure 1). Specifically, the study observed significant differences in saccular and utricular functions between healthy elderly people and those with AD and MCI (Harun et al., 2016; Wei et al., 2019). Notably, AD patients with vestibular impairment had disproportionate spatial cognitive impairment compared to AD patients without vestibular loss. The finding could imply a more specific link between the vestibular and spatial cognitive functions. It is also reported that the peripheral vestibular system could make a projection of cortical center functions, particularly those on the hippocampus involved in spatial memory and orientation (Kravitz et al., 2011; Agrawal et al., 2020) (refer to <3-2> and <3-3> with corresponding green arrows in Figure 1). In addition, older adults with vestibular impairment demonstrated poorer performance on visuospatial tests, while their vestibular function was not associated with executive function or verbal memory skills (Smith, 2017). Similarly, another research also revealed the potential link between vestibular and cognitive functions through a visual search task (Shaikh et al., 2020; Smith et al., 2020). The study found that when galvanic vestibular stimulation (GVS) was applied, the participants took a shorter time in the visual search task that required spatial memory skills (refer to <3-4> with a corresponding green arrows in Figure 1). This indicates that the stimulated vestibular function would contribute to the improvements in spatial memory skills. Overall, these results suggest that vestibular dysfunction could be an independent risk factor for dementia and the vestibular system is possibly linked with the hippocampus which is, in turn, involved in spatial memory. Considering that postural control is maintained through the interactions between visual, vestibular, and proprioceptive systems (Ivanenko and Gurfinkel, 2018; Shaikh et al., 2020), the performance of body balance could theoretically signify cognitive function. Interestingly, with the hypothesis of the potential connection between the vestibular and cognitive functions, a study evaluated the postural sway of people with MCI and AD (Ide et al., 2022). The research revealed that the index of postural stability (IPS), which is calculated by sway areas at five different body tilts, could differentiate the two groups in a specific test condition with eyes closed and with a hard surface on a stabilometer. The results also showed a significant positive correlation between the IPS and the betweenness centrality in the hippocampus derived from Magnetic Resonance Imaging (MRI), suggesting the connections between the vestibular function and the hippocampus (refer to <3-5> with corresponding green and yellow arrows in Figure 1). Therefore, body balance assessment may be useful for dementia screening in its potential to evaluate the cognitive function through the vestibular function (Cronin et al., 2017).

Looking into the possible connection between the visual and vestibular systems, the researchers observed the effect of HMD-based VR visual stimuli on the vestibular function, finding that visual stimuli displayed in a VR headset provoked the vestibular system (refer to <3-6> with corresponding blue and green arrows in Figure 1) (Ashiri et al., 2020). The research measured the vestibular activity of the subjects in two different test conditions while they were sitting on a chair. The chair was physically tilted forwards and backwards without a VR environment in the first test condition. In the second condition, the same tilting situation was simulated in a VR environment using HMD-VR technology without any physical movement in the real world. The results showed that the vestibular system responded in both conditions, yet the response was smaller in the case of simulated tilting conditions in the VR environment. Nevertheless, the study revealed the potential of using HMD-based VR environments to assess vestibular function in a more convenient and accessible manner. Thus, HMD-VR technology is expected to function as a stimulation tool to elicit vestibular activities through visual information.

To summarize the findings of previous studies explained above (refer to Figure 1), there may be a comprehensive link between the visual, vestibular, and cognitive functions theoretically, when visual stimulation is applied using HMD-VR technology. Different reactions could appear on postural sway behaviors. We have formulated our main research hypothesis that when visual stimuli of a moving VR environment are implemented into body balance assessment, the cognitive functioning could be provoked indirectly through the indirect stimulation of the vestibular function and be detected through postural sway behaviors. To evaluate this hypothesis, we have upgraded our combined assessment system of stabilometer and VR headset based on our previous study (Imaoka et al., 2020a). Specifically, the prior study did not design the VR environments considering the effect of VR visual stimuli on frequency domain postural sway data. Since the frequency response of body sway explains the movement from another angle (Jurkojc et al., 2021), the present study describes and explores newly designed VR environments that additionally evaluate specific effects of frequency-based VR visual stimuli next to the time domain.

In the present study, our long-term aim is to develop a novel comprehensive assessment system for dementia screening in a simple, low-cost, and time-efficient manner for clinical settings. As a first step, we have been concurrently analyzing postural and eye movements with HMD-VR technology. To the best of our knowledge, this is the first study to investigate the effects of visual stimuli produced by HMD-VR technology on postural sway and the possible link between cognition and body balance.

## 2. Research methods

### 2.1. Study design

This study was conducted at a local university from January 2020 to November 2021. The local ethical committee approved the ethics (2019-N-181). Older adults aged 60 years or above were recruited. First, the experimental protocol was explained to the participants. If the participants agreed to join the measurement, they were asked to sign an informed consent and answer a custom-made health questionnaire to perform an initial screening on physical impairment and motion sickness. If this first screening was successful and neither physical impairment nor motion sickness was detected, their cognitive function was accessed using the Montreal Cognitive Assessment (MoCA) and compared to the normative values that are adjusted based on age, sex, years of education, and MoCA score (Thomann et al., 2018). This study is all but the first step toward our final goal of delivering the newly developed assessment system to clinical settings. Following the guideline to develop a diagnostic test, the present study focused on evaluating the system validity in a small cohort of older adults to understand whether the proposed tests can measure what is intended to be measured (Boutros and Arflken, 2007; Colli et al., 2014; Leeflang and Allerberger, 2019). In the future experiment, specific tests will be selected to evaluate the sensitivity and specificity of the proposed assessment system in an increased sample size.

### 2.2. Experimental protocol

**Figure 2:**
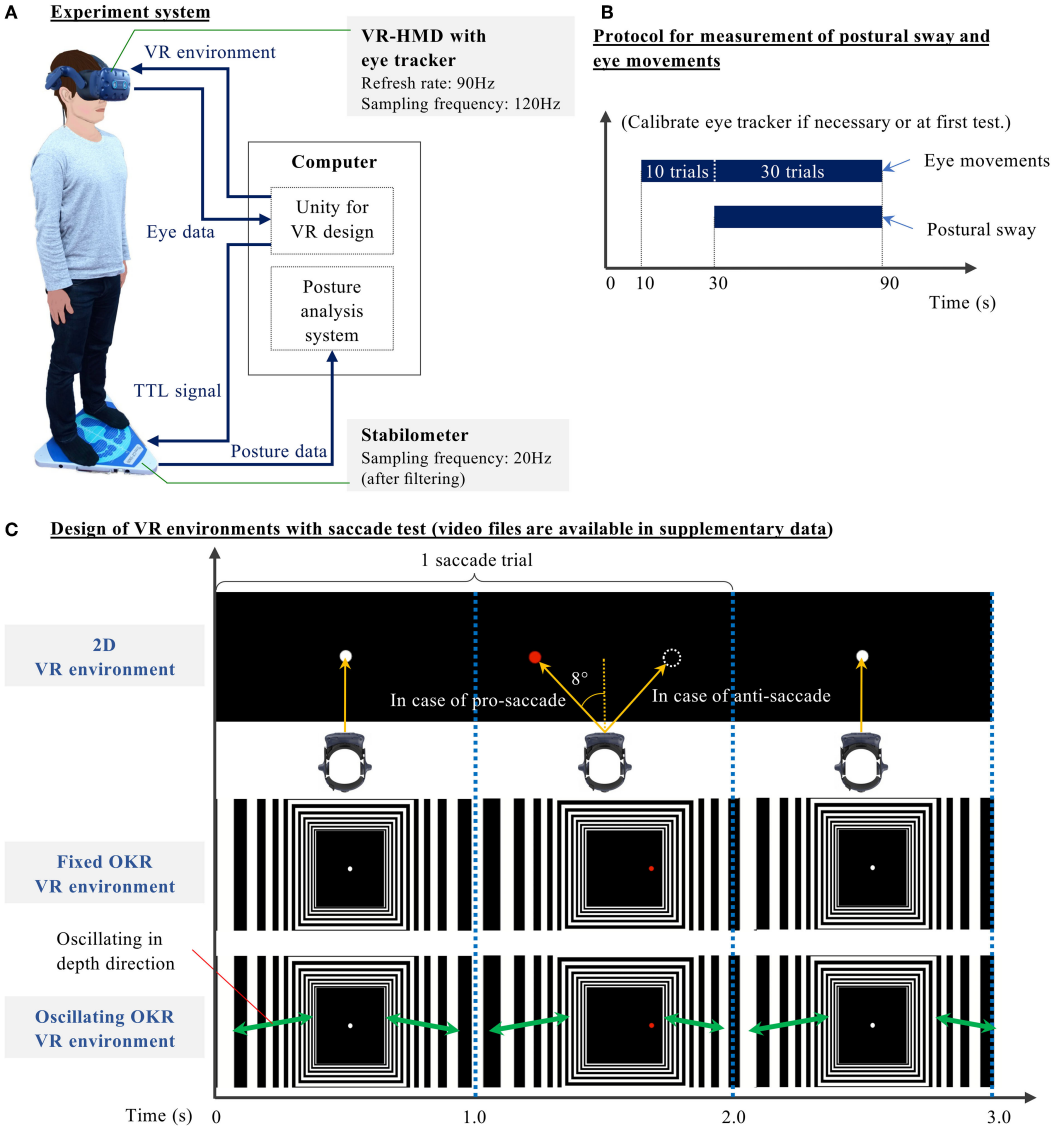
**(A)** Block diagram of the novel concurrent comprehensive assessment system of posture and eye movements using VR-HMD—Stabilometer and analysis software: GP-5000 (ANIMA Corp., Japan), VR-HMD: VIVE Pro Eye (HTC Corp., Taiwan), Computer: NUC8I7HVK (Intel Corp., U.S.), Unity: 2019.2.5f1, Eye tracker: SRanipal 1.1.0.1, SR Runtime 1.1.2.0, and Steam VR 1.11.11. **(B)** Measurement protocol of posture and eye movements—1) Stand on the stabilometer, wearing the headset; 2) Look at the black screen in the VR for initial 10 s; 3) In tests without saccade, gaze at a white central target in the VR between 10 and 90 s. In tests with a saccade, perform saccadic eye movements for 40 trials (Imaoka et al., 2020b). (Postural sway is measured between 30 and 90 seconds without notice to the participants.) **(C)** Three VR designs were developed in this study: 2D, Fixed optokinetic design-based room with stripes (OKR), Oscillating OKR—The virtual room oscillates in depth direction for 90 s in an Oscillating OKR VR environment. In conditions with the saccade test, participants gaze at a white central target for 1 s. Once the white target disappears, the red target appears on either the left or right side at 8°. Participants move their eyes toward (for pro-saccade) or opposite of (for anti-saccade) the red target within 1 s once the red target appears. The white central target is continuously displayed in the VR for 90 s in the conditions without saccade tests.

Figure 2A explains the system diagram of the novel concurrent comprehensive assessment system measuring postural sway and eye movements. The system consists of a stabilometer GP-5000 (ANIMA Corporation, Japan), which measures the displacement of the center of pressure (COP), and the VR headset VIVE Pro Eye (HTC Corporation, Taiwan), which outputs VR environments and records eye movements. This comprehensive assessment system is developed based on our previous studies that evaluated the effects of VR visual flow on postural sway speed in healthy young and older adults (Imaoka et al., 2020a) and investigated the potential of using the VIVE Pro Eye VR headset as an assessment tool of saccadic eye movement (Imaoka et al., 2020b). In the present study, the stabilometer and eye-trackers of the VR headset are synchronized on the Unity software platform to achieve the concurrent comprehensive assessment of postural sway and eye movements in diverse VR environments. We developed VR designs on the Unity platform. While showing VR environments in the VR headset, we recorded eye movements by eye-trackers embedded in the VR headset and postural sway by the stabilometer.

As described in Table 1, we prepared 11 test conditions. First, only postural sway with eyes-open (EO) condition was measured without the VR headset. The participants were asked to gaze at a cross mark at eye height and 1.5 m ahead for 60 s. Afterward, the same procedure was repeated for the eyes-closed (EC) condition without the VR headset. These non-VR conditions were prepared to evaluate the effect of VR headset weight. Subsequently, the participants conducted three different oculomotor tasks: gaze, pro-saccade, and anti-saccade in three different VR environments: only black background (2D environment), a fixed optokinetic design-based room with stripes (Fixed OKR environment), and oscillating optokinetic design-based room with stripes (Oscillating OKR environment). For the conditions with VR environments, each test took 90 s. We prepared the gaze task to make a single-task condition with only postural control and implemented the saccade tasks to build a dual-task paradigm requiring both postural and ocular motor controls. During the measurement, we asked the participants to stand on the stabilometer with their feet set wide to remove the possible influence of lower-limb muscle strength on postural sway (Imaoka et al., 2020a). The foot position was marked on the stabilometer with tapes so that the same foot position was maintained for each condition. The participants were asked to check whether they could see a sample VR environment clearly and to wear glasses or contact lenses if needed before we conducted the experiment. In the conditions with saccade measurement, the participants started with calibration of eye-trackers if necessary or in the first test and then executed the saccade tasks for 40 trials, initial 10 of which were practice trials unknown to the participants and were also prepared to eliminate the technical sampling issues (Imaoka et al., 2020b). After the initial 10 trials were performed, the Unity platform sent a trigger signal to the stabilometer to start measuring the postural sway for 60 s. The remaining 30 saccade trials were continuously measured by the eye-trackers and were used for the data analysis (refer to Figure 2B). The same measurement protocol was also applied to the VR conditions without the saccade assessment. The order of the last 9 tests was randomized. The participants took a break if necessary between the trials.

**Table 1:** Experimental conditions for the comprehensive assessment of postural sway and eye movements.

**Test**		**VR environment**		**Task**	
**#**	**Name**	**Design**	**Visual stimuli**	**Posture control**	**Saccade control**
1	Eyes-open (EO)	None	None	◯	None
2	Eyes-closed (EC)	None	None	◯	None
3	2D Gaze (2D-G)	2D	None	◯	None
4	2D Prosaccade (2D-P)	2D	None	◯	◯
5	2D Antisaccade (2D-A)	2D	None	◯	◯
6	Fixed OKR Gaze (FixOKR-G)	Fixed OKR	None	◯	None
7	Fixed OKR Prosaccade (FixOKR-P)	Fixed OKR	None	◯	◯
8	Fixed OKR Antisaccade (FixOKR-A)	Fixed OKR	None	◯	◯
9	Oscillating OKR Gaze (OscOKR-G)	Oscillating OKR	◯	◯	None
10	Oscillating OKR Prosaccade (OscOKR-P)	Oscillating OKR	◯	◯	◯
11	Oscillating OKR Antisaccade (OscOKR-A)	Oscillating OKR	◯	◯	◯

Three different VR designs were prepared: 1) 2D with a black background, 2) Fixed optokinetic design-based room with stripes (Fixed OKR), and 3) Oscillating optokinetic design-based room with stripes (Oscillating OKR). We define the oscillating VR environment as a condition to create visual stimuli (refer to Formula 1). #1 and #2 are non-VR conditions. #3 - #11 are VR conditions. OKR, OptoKinetic design-based Room; Osc, Oscillation.

Figure 2C illustrates the VR environments designed in this study. The images in the figure show an example with a saccade test (refer to Supplementary Video files for the animation of designed VR environments). First, the 2D environment consisted of a black background with targets. For the conditions without saccade measurement, the participants were asked to gaze at a white central target appearing in the VR environment for 90 s. When the participants performed saccadic eye movements, they looked at only the black screen in the VR environment for the first 10 s and then started the saccade task. Each saccade trial consists of two video frames for 2 s: 1) black background with a white central target for 1 s and 2) black background with a red target appearing horizontally on the either left or right side at 8° from the center for 1 s. The participants were asked to move their eyes toward the red target as quickly as possible in the pro-saccade task once the red target appeared in the VR environment. In the anti-saccade task, we asked the participants to move their eyes toward the opposite direction of the red target. The saccade measurement was conducted for 40 trials in total for 80 s. Next, to evaluate our main hypothesis that the cognitive function could be detected through postural sway behaviors when visual stimuli are applied in VR-HMD, we implemented the optokinetic design-based room environment because the optokinetic design is likely to stimulate vestibular function (Pavlou, 2010). Specifically, the moving room paradigm was realized in the VR environment (Jurkojc et al., 2021). The system configuration requires less space compared to the experimental system that uses a physically moving room in the reality. Thus, the proposed HMD-VR based system can be more practical in clinical settings. We designed the optokinetic design-based room environment using multiple rectangular black and white stripes that extended in depth direction. This virtual room did not move in the fixed optokinetic design-based room with stripes (Fixed OKR) VR environment, whereas it oscillated linearly in depth direction (forwards and backwards) at the frequency of 23/60Hz with sinusoidal wave (refer to Formula 1) for 90 s in the oscillating optokinetic design-based room (Oscillating OKR) VR environment (Laurens et al., 2010). We defined the Oscillating OKR VR environment as a condition to create VR visual stimuli. The linear oscillation theoretically induces the activities of the saccule and utricle in the vestibular system, where people with AD and MCI showed impairment (Wei et al., 2019; Agrawal et al., 2020; Shaikh et al., 2020). This design also provokes less motion sickness (Chang et al., 2020). In addition, we selected the specific frequency for the oscillation to mitigate the possible risk of inducing motion sickness (Bertolini and Straumann, 2016; Chang et al., 2020), while the frequency was still in the range that was considered to be related to visual and vestibular systems (Gilfriche et al., 2018). The measurement protocol in the Fixed and Oscillating OKR VR environments was the same as in the 2D VR environment. The participants followed the same procedures of eye movements as in the conditions with the 2D VR environment explained above.


(1) $$\begin{array}{l} \begin{array}{c} f\left(t\right)=0.25\cdot \sin\left(2\cdot \pi \cdot f_{c}\cdot t\right)\\ f_{c}\;:\text{ oscillating frequency}\;23/60\text{ Hz}\\ \text{t }:\text{ time in seconds} \end{array} \end{array}$$

### 2.3. Signal processing

The raw data of postural sway were processed using MATLAB R2019b (MathWorks, U.S.). The Center of pressure (COP) was measured in each medio-lateral (ML) and anterior-posterior (AP) direction. In addition to the time domain parameter of mean sway speed (Formula 2), we calculated the frequency response of postural sway up to the frequency of 10Hz, by applying zero padding of size four and Fast Fourier Transform (FFT) to the data on the time domain. We divided the whole power spectra into bins with each width of 0.05Hz. As explained in Formula 3, the normalized power spectrum was calculated in each of three frequency bands: 0 − 0.5Hz (Band 1, more associated with visual and vestibular systems Gilfriche et al., 2018), 0.5 − 2.0Hz (Band 2), and 2.0 − 10Hz (Band 3, more associated with proprioceptive system Gilfriche et al., 2018) in each sway direction to evaluate the power distribution. The power spectrum was normalized to the total signal power in each sway direction in each test condition in each participant.


(2) $$\begin{array}{l} \vec{v_{x}}=\frac{d\vec{x}}{dt}\;\vec{v_{y}}=\frac{d\vec{y}}{dt}\\ \bar{v_{x}}=\frac{1}{N-1}\;\cdot \;\sum_{i=1}^{N-1}\left(\vec{v_{x}}\right)=\frac{1}{N-1}\sum_{i=1}^{N-1}\left(|x_{i+1}-x_{i}|\;\cdot \;F_{bs}\right)\\ \bar{v_{y}}=\frac{1}{N-1}\;\cdot \;\sum_{i=1}^{N-1}\left(\vec{v_{y}}\right)=\frac{1}{N-1}\sum_{i=1}^{N-1}\left(|y_{i+1}-y_{i}|\;\cdot \;F_{bs}\right) \end{array}$$

*x*_*i*_, *y*_*i*_ : COP in each ML and AP direction at *i*th sample in *j*th participant
*N* : Total number of samples
Displacement of COP : $\vec{\text{x}}$ = [*x*_1_, *x*_2_, …, *x*_*i*_, …, *x*_*N*_] (ML direction),
$\vec{\text{y}}$ = [*y*_1_, *y*_2_, …, *y*_*i*_, …, *y*_*N*_] (AP direction)
$\bar{v_{x}}$, $\bar{v_{y}}$ : Mean sway speed in each ML and AP direction
*F*_*bs*_ : Sampling frequency for postural sway (20Hz)

$$\begin{array}{l} \begin{array}{c} \vec{\text{X}}=\left[\begin{array}{c} \vec{X_{1}}\\ \vdots \\ \vec{X_{j}}\\ \vdots \\ \vec{X_{N_{p}}} \end{array}\right]=\left[\begin{array}{ccccc} X_{1,1} & \cdots & X_{1,m} & \cdots & X_{1,N_{bin}}\\ \vdots & \ddots & \vdots & \ddots & \vdots \\ X_{j,1} & \cdots & X_{j,m} & \cdots & X_{j,N_{bin}}\\ \vdots & \ddots & \vdots & \ddots & \vdots \\ X_{N_{p},1} & \cdots & X_{N_{p},m} & \cdots & X_{N_{p},N_{bin}} \end{array}\right]\\ \vec{\text{Y}}=\left[\begin{array}{c} \vec{Y_{1}}\\ \vdots \\ \vec{Y_{j}}\\ \vdots \\ \vec{Y_{N_{p}}} \end{array}\right]=\left[\begin{array}{ccccc} Y_{1,1} & \cdots & Y_{1,m} & \cdots & Y_{1,N_{bin}}\\ \vdots & \ddots & \vdots & \ddots & \vdots \\ Y_{j,1} & \cdots & Y_{j,m} & \cdots & Y_{j,N_{bin}}\\ \vdots & \ddots & \vdots & \ddots & \vdots \\ Y_{N_{p},1} & \cdots & Y_{N_{p},m} & \cdots & Y_{N_{p},N_{bin}} \end{array}\right] \end{array} \end{array}$$

$\vec{\text{X}}$, $\vec{\text{Y}}$ : Power spectra of all participants in each ML and AP direction after being divided into bins
*N*_*p*_ : Number of participants
*N*_*bin*_ : Number of frequency bins
*X*_*j,m*_, *Y*_*j,m*_ : Power spectrum at *m*th bin in *j*th participant in each ML and AP direction

$$\begin{array}{l} P_{bq,X}=\sum_{m=N_{qs}}^{N_{qe}}\left(X_{j,m}\right)/\sum_{m=1}^{N_{bin}}\left(X_{j,m}\right)\\ P_{bq,Y}=\sum_{m=N_{qs}}^{N_{qe}}\left(Y_{j,m}\right)/\sum_{m=1}^{N_{bin}}\left(Y_{j,m}\right) \end{array}$$

*P*_*bq,X*_ : Normalized power spectrum of frequency band *q* in ML direction in *j*th participant
*P*_*bq,Y*_ : Normalized power spectrum of frequency band *q* in AP direction in *j*th participant
*q* : Order of frequency band (*q* = 1, 2, 3), Band 1 for 0 − 0.5Hz, Band 2 for 0.5 − 2.0Hz, Band 3 for 2.0 − 10Hz
*N*_*qs*_ : Order of first frequency bin in frequency band *q*
*N*_*qe*_ : Order of last frequency bin in frequency band *q*

### 2.4. Statistical analysis

The processed data were statistically analyzed on R programming with version 4.0.2 (R Core Team, 2020).

#### 2.4.1. Check of statistical test requirements

Levene's test and Shapiro-Wilk test were conducted to check the homogeneity of variance and the normality respectively for the following parameters: mean sway speed ($\bar{v_{x}}$, $\bar{v_{y}}$) and normalized power spectrum of frequency bands (*P*_*b*1,*X*_, *P*_*b*1,*Y*_, *P*_*b*2,*X*_, *P*_*b*2,*Y*_, *P*_*b*3,*X*_, *P*_*b*3,*Y*_) of postural sway.

#### 2.4.2. Effects of VR visual stimuli

After inspecting the normality and homogeneity of variance for each measurement parameter, we applied non-parametric analysis of variance (ANOVA) to the VR test conditions (#3–#11 in Table 1) to investigate the effects of VR visual stimuli on posture (5% significance level) (Noguchi et al., 2012). We focused on these VR conditions to eliminate the possible effect of the VR headset weight on body sway. For *post-hoc* analyses, Wilcoxon signed-rank test was performed to evaluate the *P*-values and effect sizes (*ES*), by comparing each measurement parameter between the test conditions. We adjusted the calculated *P*-values because of multiple *post-hoc* pairwise comparisons (Benjamini and Hochberg, 1995).

#### 2.4.3. Association between cognitive function and postural sway

We evaluated the relationship between the MoCA z-score derived from the formula (Thomann et al., 2018) and postural sway parameters: mean sway speed and power spectrum of each frequency band in each test condition. We calculated non-parametric Spearman's rank correlation coefficient: *ES* ≥ 0.5: large, 0.5 ≥ *ES* ≥ 0.3: moderate, 0.3 ≥ *ES* ≥ 0.1: small.

## 3. Results

### 3.1. Participants

After our initial screening with the health questionnaire, 23 older adults joined the experiment. Table 2 shows the demographic profile of the participants. No participants reported motion sickness before or during the measurement. The MoCA screening found that 3 participants did not achieve the cutoff of the MoCA z-score, which was derived from the normative data (Thomann et al., 2018).

**Table 2:** Demographic profile of participants in the experiment.

**Variables**	**Healthy group**	**MCI group**	***P*-value**
	**(*N* = 20)**	**(*N* = 3)**	
Sex	Men: 11, Women: 9	Men: 1, Women: 2	
Age	70.4 ± 4.9 years	74.7 ± 4.0 years	0.14
Weight	85.2 ± 22.6 kg	69.6 ± 7.5 kg	0.23
Height	172.0 ± 7.9 cm	170.0 ± 2.0 cm	0.55
BMI	28.4 ± 5.8 kg/m^2^	24.1 ± 2.3 kg/m^2^	0.17
Education	13.9 ± 2.9 years	14.0 ± 1.7 years	0.96
MoCA score	27.2 ± 1.6	20.3 ± 2.1	0.006^**^
MoCA z-score	0.2 ± 0.8	−2.3 ± 0.2	0.001^***^

** indicates *P* ≤ 0.01,

*** indicates *P* ≤ 0.001.

### 3.2. Normality and homogeneity of variance of measurement parameters

The results of Levene's test and Shapiro-Wilk test showed that postural parameters need to be analyzed, using non-parametric statistics in the following analyses.

### 3.3. Non-parametric ANOVA for postural sway in all test conditions

Table 3 shows the *P*-values calculated by non-parametric ANOVA to evaluate the effect of different visual stimuli on postural sway. The main effect was seen for postural sway in the anterior-posterior direction for all the parameters (*P* < 0.001). On the other hand, the effect was not observed for body sway in the medio-lateral direction (*P* ≥ 0.23).

**Table 3:** Results of non-parametric ANOVA for postural sway parameters.

**Parameters**	***P*-values**
Mean sway speed - ML	0.31
Mean sway speed - AP	< 0.001^***^
Power spectrum band 1 - ML	0.27
Power spectrum band 1 - AP	< 0.001^***^
Power spectrum band 2 - ML	0.23
Power spectrum band 2 - AP	< 0.001^***^
Power spectrum band 3 - ML	0.26
Power spectrum band 3 - AP	< 0.001^***^

(Frequency band 1: 0−0.5Hz, band 2: 0.5−2.0Hz, band 3: 2.0−10Hz, ML, medio-lateral; AP, anterior-posterior).

*** indicates *P* ≤ 0.001.

### 3.4. Postural sway on time domain

**Figure 3:**
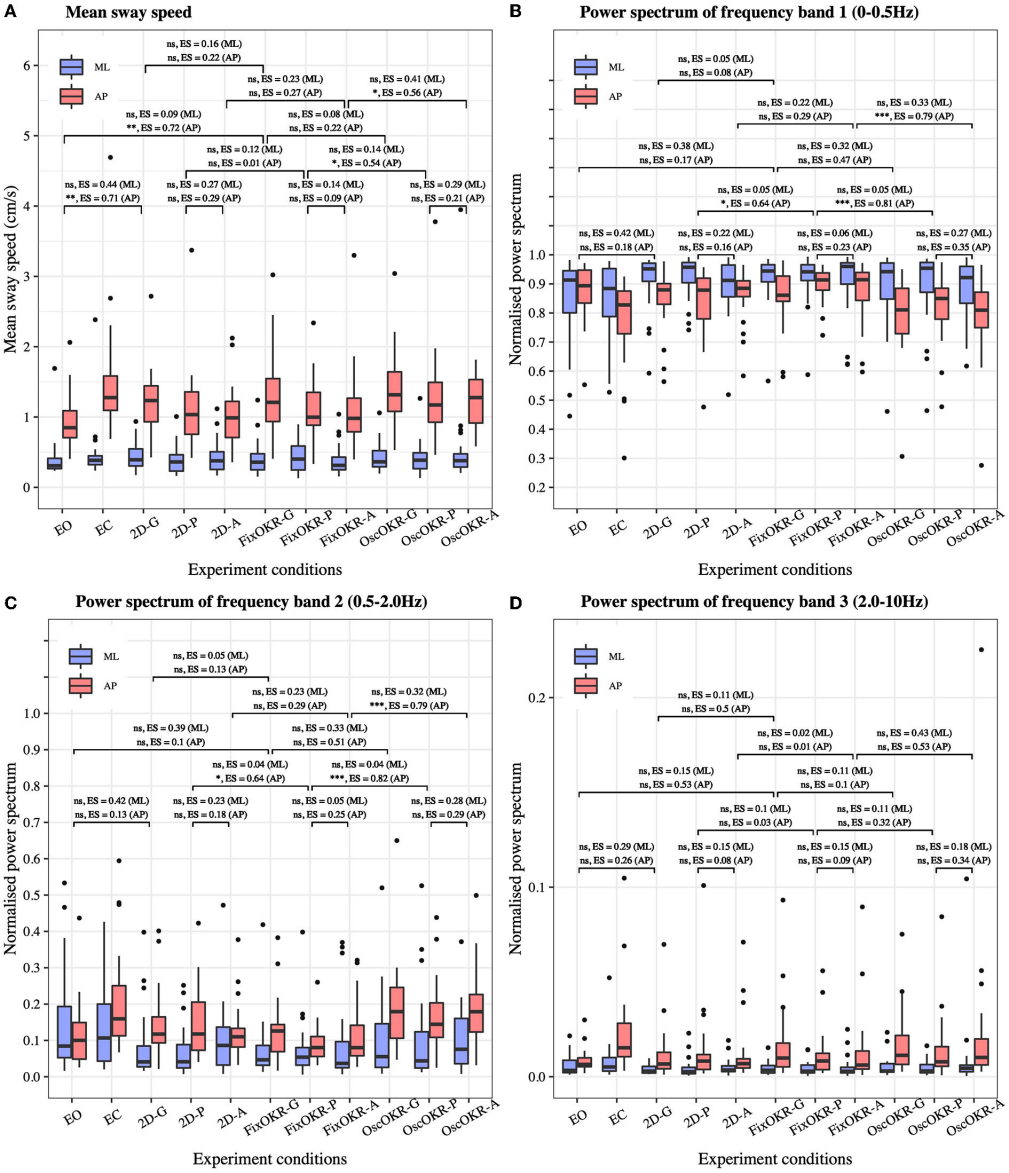
Postural sway in each test condition. **(A)** Mean sway speed. **(B)** Power spectrum of frequency Band 1: 0 − 0.5Hz (related to the visual and vestibular systems). **(C)** Power spectrum of frequency Band 2: 0.5 − 2.0Hz. **(D)** Power spectrum of frequency Band 3: 2.0 − 10Hz (related to the proprioceptive system). The effect of Oscillating OKR was observed in mean sway speed and power spectrum of frequency Band 1 and Band 2 in anterior-posterior direction when saccade task was also performed (OscOKR-P and OscOKR-A). The dual-task of posture and the saccade controls could cause more postural sway possibly due to the limited capacity of resources for the two movements. The OKR oscillating at 23/60Hz did not influence the proprioceptive function. (-G, Gaze; -P, Pro-saccade; -A, Anti-saccade; OKR, OptoKinetic design-based Room with stripes; FixOKR, Fixed OKR; OscOKR, Oscillating OKR).

Figure 3A illustrates the distribution of mean postural sway speed in each medio-lateral and anterior-posterior direction of all the participants. No significant differences were seen in medio-lateral direction between eyes-open and 2D Gaze conditions (*P* = 0.11, *ES* = 0.44) and between 2D Gaze and Fixed OKR Gaze conditions (*P* = 0.69, *ES* = 0.16). On the other hand, we observed significant differences in body sway in anterior-posterior direction between eyes-open and 2D Gaze conditions (*P* = 0.004, *ES* = 0.71), but not between 2D Gaze and Fixed OKR Gaze conditions (*P* = 0.52, *ES* = 0.22). Comparing the sway performance between the test conditions with the oscillation and those without it, we did not find a significant difference between Fixed OKR Gaze and Oscillating OKR Gaze conditions (*P* = 0.81, *ES* = 0.08 in medio-lateral direction; *P* = 0.52, *ES* = 0.22 in anterior-posterior direction). However, the results showed significant differences in anterior-posterior body sway between Fixed OKR Pro-saccade and Oscillating OKR Pro-saccade conditions (*P* = 0.73, *ES* = 0.14 in medio-lateral direction; *P* = 0.03, *ES* = 0.54 in anterior-posterior direction) and between Fixed OKR Anti-saccade and Oscillating OKR Anti-saccade conditions (*P* = 0.14, *ES* = 0.41 in medio-lateral direction; *P* = 0.03, *ES* = 0.56 in anterior-posterior direction).

### 3.5. Postural sway on frequency domain

Figures 3B–D show the distribution of the normalized power spectrum of postural sway in each medio-lateral and anterior-posterior direction for (B) frequency Band 1 (0 − 0.5Hz), (C) frequency Band 2 (0.5 − 2.0Hz), and (D) frequency Band 3 (2.0 − 10Hz). Analyzing the results similarly to the time domain analysis, we did not find a significant difference between Fixed OKR Gaze and Oscillating OKR Gaze conditions (*P* = 0.27, *ES* = 0.32 in medio-lateral direction; *P* = 0.09, *ES* = 0.47 in anterior-posterior direction) in frequency Band 1. Nonetheless, significant differences were observed in anterior-posterior postural sway in frequency Band 1 between Fixed OKR Pro-saccade and Oscillating OKR Pro-saccade conditions (*P* = 0.87, *ES* = 0.05 in medio-lateral direction; *P* = 0.001, *ES* = 0.81 in anterior-posterior direction) and between Fixed OKR Anti-saccade and Oscillating OKR Anti-saccade conditions (*P* = 0.26, *ES* = 0.33 in medio-lateral direction; *P* = 0.001, *ES* = 0.79 in anterior-posterior direction). Similar results were found in frequency Band 2. There was no significant difference between Fixed OKR Gaze and Oscillating OKR Gaze conditions (*P* = 0.26, *ES* = 0.33 in medio-lateral direction; *P* = 0.06, *ES* = 0.51 in anterior-posterior direction), while significant differences were seen in anterior-posterior body sway between Fixed OKR Pro-saccade and Oscillating OKR Pro-saccade conditions (*P* = 0.89, *ES* = 0.04 in medio-lateral direction; *P* < 0.001, *ES* = 0.82 in anterior-posterior direction) and between Fixed OKR Anti-saccade and Oscillating OKR Anti-saccade conditions (*P* = 0.28, *ES* = 0.32 in medio-lateral direction; *P* < 0.001, *ES* = 0.79 in anterior-posterior direction). On the other hand, the results did not reveal significant differences between these test conditions in postural sway in frequency Band 3: between Fixed OKR Gaze and Oscillating OKR Gaze conditions (*P* = 0.82, *ES* = 0.11 in medio-lateral direction; *P* = 0.84, *ES* = 0.10 in anterior-posterior direction), between Fixed OKR Pro-saccade and Oscillating OKR Pro-saccade conditions (*P* = 0.82, *ES* = 0.11 in medio-lateral direction; *P* = 0.37, *ES* = 0.32 in anterior-posterior direction), and between Fixed OKR Anti-saccade and Oscillating OKR Anti-saccade conditions (*P* = 0.16, *ES* = 0.43 in medio-lateral direction; *P* = 0.07, *ES* = 0.53 in anterior-posterior direction).

### 3.6. Association between postural sway parameters and MoCA score

Table 4 shows the correlation coefficient between postural sway parameters and MoCA z-score (Thomann et al., 2018) in each test condition #1 - 11. The bold values signify the associations at a 5% significance level. A moderate association was observed between the MoCA z-score and power spectrum of the center of pressure (COP) displacement in anterior-posterior direction only for the non-saccade condition with Oscillating optokinetic design-based room with stripes (Test #9 OscOKR-G). The analysis revealed the correlation coefficients of *r*_*s*_ = 0.45 (*P* = 0.03) for the frequency Band 1: 0 − 0.5Hz, *r*_*s*_ = −0.45 (*P* = 0.03) for the frequency Band 2: 0.5 − 2.0Hz, and *r*_*s*_ = −0.25 (*P* = 0.24) for the frequency Band 3: 2.0 − 10Hz in anterior-posterior postural sway.

**Table 4:** Correlation coefficient between postural sway parameters and MoCA z-score (Thomann et al., 2018).

**Posture parameters**	**VR environment (above) and eye movements (below)**					
	**None**		**None**	**2D**	**2D**	**2D**
	**Eyes open**		**Eyes closed**	**Gaze**	**Pro-saccade**	**Anti-saccade**
Mean sway speed - ML	0.14		0.31	0.09	–0.01	–0.17
Mean sway speed - AP	–0.00		0.01	–0.06	–0.00	–0.24
Power spectrum of frequency Band 1 - ML	–0.37		–0.19	–0.03	–0.21	–0.19
Power spectrum of frequency Band 1 - AP	0.35		–0.06	0.14	0.17	0.11
Power spectrum of frequency Band 2 - ML	0.35		0.20	0.05	0.22	0.16
Power spectrum of frequency Band 2 - AP	–0.35		0.03	-0.15	–0.22	–0.11
Power spectrum of frequency Band 3 - ML	0.28		0.26	–0.10	0.24	0.07
Power spectrum of frequency Band 3 - AP	–0.25		0.20	–0.16	0.04	0
**Posture parameters**	**VR environment (above) and eye movements (below)**					
	**Fixed OKR**	**Fixed OKR**	**Fixed OKR**	**Oscillating OKR**	**Oscillating OKR**	**Oscillating OKR**
	**Gaze**	**Pro-saccade**	**Anti-saccade**	**Gaze**	**Pro-saccade**	**Anti-saccade**
Mean sway speed - ML	–0.35	–0.04	–0.25	0.01	–0.03	0.02
Mean sway speed - AP	–0.2	–0.22	–0.22	–0.04	–0.15	–0.03
Power spectra of frequency Band 1 - ML	-0.14	–0.34	–0.15	0.03	–0.01	-0.05
Power spectra of frequency Band 1 - AP	0.24	0.20	0.35	0.45^*^	0.23	-0.17
Power spectra of frequency Band 2 - ML	0.12	0.34	0.17	–0.02	0.01	0.04
Power spectra of frequency Band 2 - AP	–0.2	–0.19	–0.37	-0.45^*^	-0.26	0.14
Power spectra of frequency Band 3 - ML	0	0.09	0.11	–0.26	–0.19	0.17
Power spectra of frequency Band 3 - AP	–0.12	–0.23	–0.17	–0.25	–0.13	0.14

Significant associations were seen between the MoCA z-score and the postural sway in anterior-posterior direction: the power spectrum of anterior-posterior postural sway in frequency Band 1 and Band 2 in the oscillating condition without saccade tasks (Oscillating OKR Gaze). The HMD-based VR visual stimuli might lead the cognitive function to appear on postural sway behaviors. The similar finding was not observed when saccade task was added (Oscillating OKR Pro-saccade and Oscillating OKR Anti-saccade). The dual-task of posture and saccade controls could cause resource competition between the two movements. The sharing ratio of resource may vary depending on the participant, leading to non-significant associations. Bold values indicate correlation coefficients at 5% significance level.

* indicates *P* ≤ 0.05. ML, medio-lateral; AP, anterior-posterior; OKR, OptoKinetic design-based Room.

## 4. Discussion

### 4.1. Effects of HMD-based VR visual stimuli on postural sway

We investigated the influence of VR visual stimuli of oscillating optokinetic design-based room with stripes (Oscillating OKR) on postural sway. Non-parametric ANOVA (Table 3) revealed the main effect of different VR environments on anterior-posterior body sway in all the postural sway parameters of mean sway speed and power spectrum in each frequency band. However, we did not see significant differences in the postural sway in the medio-lateral direction. The results suggest that the changing VR environments provoked more postural sway only in the anterior-posterior direction as expected.

We further explored the effects of various VR environments in *post-hoc* analyses, including the non-VR conditions with eyes-open (EO) and eyes-closed (EC). First, we investigated the effects of wearing the VR headset, focusing on the conditions of #1 Eyes-open, #3 2D Gaze, and #6 Fixed optokinetic design-based room with stripe (OKR) Gaze. These conditions do not have the VR visual stimuli of oscillating OKR. Our results reveal that wearing the VR headset induced more mean postural sway speed in the anterior-posterior direction (Figure 3A). This implies that the test condition with VR headset and gaze task (i.e., #3 2D Gaze) should be used as a baseline condition when the effects of different VR environments are evaluated on postural sway. Second, we inspected the effect of the Oscillating OKR VR environment, by comparing the body sway data between the conditions with the oscillation and those without the oscillation: between #6 Fixed OKR Gaze and #9 Oscillating OKR Gaze conditions, between #7 Fixed OKR Pro-saccade and #10 Oscillating OKR Pro-saccade conditions, and between #8 Fixed OKR Anti-saccade and #11 Oscillating OKR Anti-saccade conditions. The results indicate that postural sway performance was influenced significantly in anterior-posterior direction by the OKR VR environment oscillating forwards and backwards if saccadic eye movements were also performed (refer to Figure 3). Specifically, the significant effect is found in the frequency Band 1: 0 − 0.5Hz, which is more related to the visual and vestibular systems (Gilfriche et al., 2018), as expected. On the other hand, the oscillation did not trigger the proprioceptive system as the significant difference is not seen in the frequency Band 3: 2.0 − 1Hz. Notably, a previous study reported that the conventional approach of postural sway measurement without VR environments showed significant differences between eyes-open and eyes-closed conditions in all the frequency ranges in anterior-posterior direction (Singh et al., 2012). Thus, the proposed new body balance assessment using HMD-based VR technology could have the potential to create new posture assessment paradigms. These results suggest that the virtual room oscillating at the frequency of *f*_*c*_ = 23/60Hz would be effective to provoke postural sway at the specific frequency range. A large effect was not observed between #6 Fixed OKR Gaze and #9 Oscillating OKR Gaze conditions probably because there was no saccade control in these test conditions. The dual-task of posture and saccade controls in the other conditions (tests #7, 8, 10, 11) could influence postural sway significantly because of the limited capacity of resources for competitive interactions between posture maintenance and saccadic eye movements (Franconeri et al., 2013) and shared attentional resources in the dual-task (Lacour et al., 2008).

To summarize, we have observed that the VR visual stimuli of an optokinetic design-based room with stripes oscillating forwards and backwards induced significant postural sway in the anterior-posterior direction in the real world, especially when the dual-task of posture and saccade controls were required. In particular, our results revealed that the virtual room oscillating at the frequency of *f*_*c*_ = 23/60Hz evoked the postural sway related to the visual and vestibular systems.

### 4.2. Association between postural control and cognitive performance

We calculated the correlation coefficients between postural sway parameters and MoCA z-score in each test condition to evaluate our primary hypothesis that postural sway behaviors stimulated by the visual stimuli of oscillating optokinetic design-based room with stripes (Oscillating OKR) could be linked with cognitive performance.

As shown in Table 4, a moderate association is found between the MoCA z-score and body sway in the anterior-posterior direction only for the condition of #9 Oscillating OKR Gaze. Specifically, the power spectrum of the center of pressure (COP) displacement in each frequency Band 1 (0 − 0.5Hz, which is more related to the visual and vestibular systems Gilfriche et al., 2018) and Band 2 (0.5 − 2.0Hz) is found to be associated with the MoCA score, while the power spectrum in the frequency Band 3 (2.0 − 10Hz, which is more linked to the proprioceptive system Gilfriche et al., 2018) does not associate with the MoCA score. This may suggest that the cognitive function would be more linked with the visual and vestibular systems than the proprioceptive system as expected. On the other hand, similar findings are not seen in the conventional approach to body balance measurement in the eyes-open (EO) and eyes-closed (EC) conditions. The results reveal that the newly designed HMD-based VR visual stimuli of Oscillating OKR could be effective to affect only the visual and vestibular systems and, thus, create a potential link between the provoked body sway and the cognitive function as hypothesized. This implies that cognitive function might be detected through body balance assessment. Interestingly, while we find the significant influence of the Oscillating OKR VR environment on postural sway in the conditions with saccade assessment as discussed above (refer to Figure 3), a weak correlation is observed between the body sway and the MoCA score in these conditions: #10 Oscillating OKR Prosaccade and #11 Oscillating OKR Antisaccade. This may indicate that the focus on a single task of posture control in the Oscillating OKR VR environment would be necessary to make a link between the cognitive function and motor function of postural sway. The inclusion of saccade control might hinder postural control due to limited attentional resources and different ratios of resource sharing between posture and saccade controls in each participant (Huxhold et al., 2006; Lacour et al., 2008). Remarkably, the better the cognitive performance is, the larger the power spectrum of postural sway gets in the frequency Band 1. In contrast, the spectrum of the frequency Band 2 gets smaller when the MoCA z-score is higher. A previous study evaluating the postural sway of healthy adults and people with vestibular dysfunction also found a significant postural instability in the group of vestibular impairment for the frequency range: 0.5 − 1.0Hz (Gorski et al., 2019). Our results may indicate that when cognitive performance deteriorates, the sensitivity of visual and vestibular systems could also decline. People with low cognitive functioning might have difficulty in adapting to the Oscillating OKR VR environment, possibly causing different postural sway behaviors in the frequency Band 1 and Band 2.

In conclusion, the single task of posture control in the OKR VR environment oscillating at the frequency of *f*_*c*_ = 23/60Hz would be effective to evoke the visual and vestibular systems, not the proprioceptive system, and generate a possible connection between the cognitive function and motor function of posture control. Lower cognitive functioning might be related to problems in maintaining postural sway in the Oscillating OKR VR environment. The results of this study may suggest that the cognitive function could be measured indirectly through body sway performance when the specific HMD-based VR visual stimuli are applied. Nevertheless, the dual-task conditions requiring posture maintenance and saccade control (#10 Oscillating OKR Prosaccade and #11 Oscillating OKR Antisaccade conditions) are not likely to create a link between the motor function of postural sway and the cognitive function. This is possibly because cognitive resources are dispersed in the two movements of body sway and saccades differently depending on the individual.

### 4.3. Limitations and future study

The main purpose of this study was to evaluate the effect of HMD-based VR visual stimuli on postural sway in older adults and to investigate whether the provoked body sway could be associated with cognitive performance. We used the novel concurrent comprehensive assessment system of posture and eye movements using HMD-VR technology. The results reveal that the visual stimuli of a virtual optokinetic design-based room with stripes (OKR VR environment) oscillating forwards and backwards at the frequency of *f*_*c*_ : 23/60Hz provoked different anterior-posterior postural sway in the real world. The MoCA z-score was found to be associated with the stimulated postural sway in frequency Band 1: 0 − 0.5Hz, which is more related to the visual and vestibular functions, and Band 2: 0.5 − 2.0Hz. Prior research suggested that 1) the vestibular function is linked with the cognitive function and 2) HMD-based VR technology could elicit the vestibular function through visual stimulation. As we hypothesized, we observed a possible link between the cognitive test performance and the provoked postural sway behaviors when the Oscillating OKR was implemented in the VR environment (#9 Oscillating OKR Gaze condition). However, we acknowledge the following limitations in this study.

First, while we hypothesized that there would be a link between the spatial cognitive function and the vestibular function when the HMD-based VR visual stimuli are applied, we did not measure these functions in real time during the measurement. Since this present study has found the potential link between cognition and postural sway, we will evaluate the vestibular and spatial cognitive functions distinctively in future research. For example, electrovestibulography (EVestG) can be a useful tool to evaluate the vestibular activity quantitatively while we measure postural sway and eye movements simultaneously (Brown et al., 2017; Ashiri et al., 2020, 2021). The measurement device can be integrated into our comprehensive assessment system as prior research also combined it with a VR headset (Ashiri et al., 2021). Similarly, incorporating an electroencephalogram (EEG) device could clarify the changes of activities in the parietal cortex in real time, which is directly related to the spatial cognitive function (Sack, 2009; Zhou et al., 2020). Adding these measures to our novel assessment system of posture and eye movements would lead us to understand the vestibular and spatial cognitive functions precisely in each VR environment. Moreover, a more comprehensive analysis system including, for instance, the following systems would further enhance the assessment capability and thus elucidate further the underlying mechanisms of posture: sophisticated eye-trackers to achieve the level of ocular assessment with ophthalmoscopy and electromyography (EMG) sensors to measure muscle activities in the additional condition with physical perturbation from the stabilometer.

Second, we did not measure each of the visual, vestibular, and proprioceptive functions separately that are considered to contribute to postural control (Ivanenko and Gurfinkel, 2018). For example, there are multiple factors affecting visual function such as visual acuity, contrast sensitivity, and perceptions of colors, depth, and motions (Bennett et al., 2019). A more detailed assessment of the visual function using ophthalmoscopy could help us understand the function independently and precisely. The separate assessment of visual function could be also used as another potential biomarker for the dementia screening (Salobrar-Garcia et al., 2016). The vestibular function can be also measured individually based on the vestibulo-ocular reflex (VOR), by physically stimulating the vestibular system with various methods such as caloric reflex test and rotational chair with eye-trackers (Fife et al., 2000; Miles and Zapala, 2015). Similarly, while there are diverse methods of investigating the proprioceptive function (Hillier et al., 2015), measuring muscle activities of lower limbs using EMG in a condition with physical perturbation could elucidate the function in a simple way. Thus, if we evaluate each of the visual, vestibular, and proprioceptive functions individually and use the data as a baseline, we could enhance the entire analysis of postural sway behaviors by clarifying more detailed links between the underlying functions and the HMD-based VR visual stimuli.

Third, along with the limitations mentioned above, the effect of VR environments designed to provoke behavioral reactions needs to be further inspected. We designed the VR scenery based on prior studies that found activated vestibular function by visual stimuli in HMD-based VR environments (Ashiri et al., 2020) and reported the potential activation of saccule and utricle by linear oscillation (Wei et al., 2019; Agrawal et al., 2020; Shaikh et al., 2020). However, the VR designs in this present study are not the same as those used in some other prior research and our experiment did not measure the vestibular function. Therefore, future studies need to uncover the activities of vestibular function while displaying different types of VR environments.

Fourth, the changes of postural sway observed during the measurement with the HMD-based VR visual stimuli could be simply due to aging. While it should be also noted that postural instability is associated with waning cognitive ability (Sullivan et al., 2009), previous studies also found more declining postural stability as the population ages (Sullivan et al., 2009; Degani et al., 2017). Moreover, research evaluating postural sensitivity to visual flow also found that older adults with balance problems revealed greater visual sensitivity and greater use of a hip postural strategy when they reacted to the moving visual flow, in comparison with young adults and older adults without balance issues (Sundermier et al., 1996). To exclude the people with motor dysfunction affecting their postural sway performance, we screened the participants before the measurement by checking their physical condition using a health questionnaire. Nonetheless, the screening might not be sensitive enough to detect small balance problems accurately. Some participants in this study could also have different visual sensitivity from the others, generating different postural sway behaviors. In future research, we will enhance the screening process by investigating the postural sway performance objectively. For instance, we would use Romberg's test to inspect body sway in eyes-open and eyes-closed conditions without the VR headset (Forbes and Cronovich, 2022) or postural sensory organization test (Clendaniel, 2000). Moreover, our study prepares a baseline test condition without the VR visual stimuli. We will compare the postural sway data between the test conditions with and without the visual stimuli in each participant. The comparison could potentially cancel the effect of normal aging in each participant and focus on only the effect of VR visual stimuli.

Fifth, while we used the MoCA score to inspect the relationship between cognition and posture, the score may not always show the spatial cognitive ability because the MoCA assesses various functions: visuospatial and executive functions, short-term memory, attention, animal naming, language, abstraction, delayed recall, and orientation (Nasreddine et al., 2005). Adding specific spatial cognitive tests such as block design, clock drawing, and Rey-Osterrieth complex figure, which are potentially useful for the diagnosis of AD (Salimi et al., 2017), will enable us to understand the baseline spatial cognitive function more precisely and enhance the correlation analysis. In addition, integrating spatial navigation tasks in our assessment battery with HMD-VR technology could be an alternative to measure the spatial cognitive function (Cogne et al., 2017).

Finally, the sample of this study was more healthy older adults. Our future study needs to increase the population with cognitive impairment and investigates whether HMD-based VR visual stimuli would be effective to observe differences in postural sway behaviors between healthy elderly people and those with cognitive impairment.

In summary, this present study is the first step to evaluating the effects of HMD-based VR visual stimuli on postural sway behaviors and the relationship between the stimulated postural sway and cognitive function. In future research, extending the scope of the newly developed comprehensive assessment system with integrated EVestG and EEG devices will be important to inspect the findings of this study more specifically. A more detailed assessment of the visual, vestibular, and proprioceptive functions for posture and the spatial cognitive function is also important to enhance the whole study by clarifying the baseline status of each function. Eventually, research with more sample size of people with MCI and dementia will be critical to evaluate the validity of the proposed assessment system.

## 5. Conclusion

In our growing aging society, dementia is globally becoming a relevant problem and early dementia screening will be important to provide more intervention options at an early stage. Assessing postural sway behaviors using a head-mounted display (HMD) virtual reality (VR) technology could be useful as a simple tool for dementia screening. Following prior research findings that 1) vestibular impairment is strongly associated with cognitive dysfunction in people with mild cognitive impairment (MCI) and Alzheimer's disease (AD) and 2) visual stimuli in HMD-based VR environments provoked the vestibular function, we have hypothesized that there would be an association between the cognitive function and postural sway when HMD-based VR visual stimuli are applied. We evaluated the effect of VR visual stimuli on posture and the relationship between the stimulated body sway and cognitive performance in older adults. Our results show that the visual stimuli of an optokinetic design-based room with stripes (OKR) VR environment oscillating forwards and backwards at the frequency of 23/60Hz induced different postural sway in the anterior-posterior direction, but not in the medio-lateral direction, in the real world. Specifically, our frequency response analysis of the center of pressure (COP) displacement reveals that the visual and vestibular systems were likely to be affected (frequency Band 1: 0 − 0.5Hz), while the proprioceptive system was not evoked (frequency Band 3: 2.0 − 10Hz). The correlation analysis finds a moderate association between the cognitive performance and the stimulated postural sway performance in a single task condition of posture control in the Oscillating OKR VR environment. These findings would suggest that the potential link may appear between the cognitive function and postural sway when the HMD-based VR visual stimuli are applied. The results of this first step study warrant further research, 1) integrating into our novel comprehensive assessment system the measures to investigate the vestibular and spatial cognitive functions simultaneously and specifically, and 2) including individual assessment of spatial cognitive ability and visual, vestibular, and proprioceptive systems for posture control. These improvements would further elucidate the possible link between cognition and posture stimulated by HMD-based VR technology. Assessing a larger sample size of older adults with cognitive impairment could improve the validity of the proposed novel assessment system using HMD-based VR technology.

## Data availability statement

The raw data supporting the conclusions of this article will be made available by the authors, without undue reservation.

## Ethics statement

The studies involving human participants were reviewed and approved by ETH Zurich Ethics Commission. The patients/participants provided their written informed consent to participate in this study.

## Author contributions

Each of the authors has contributed to developing the research concept and experimental designs. YI and AF developed the concurrent assessment system and designed VR environments. YI, AF, and LH performed the measurement and analyzed the data. All authors interpreted the analyzed data, contributed to drafting, revising the article to bring it to its current state, and approved the final version to be published.

## Funding

Open access funding provided by ETH Zurich.

## Conflict of interest

The authors declare that the research was conducted in the absence of any commercial or financial relationships that could be construed as a potential conflict of interest.

## Publisher's note

All claims expressed in this article are solely those of the authors and do not necessarily represent those of their affiliated organizations, or those of the publisher, the editors and the reviewers. Any product that may be evaluated in this article, or claim that may be made by its manufacturer, is not guaranteed or endorsed by the publisher.

## Supplementary material

- Video file of 2D VR environment with a task of saccadic eye movement.
- Video file of Oscillating optokinetic design-based room with stripes (Oscillating OKR) VR environment with a task of saccadic eye movement.
- Basic source code and 2D VR design for saccade assessment using HTC VIVE Pro Eye VR headset: https://github.com/MotorControlLearning/SaccadeVR-mobile.
